# Bis{4-[(*Z*)-(4-fluoro­benzyl­amino)(phenyl)­methyl­ene]-3-methyl-1-phenyl-1*H*-pyrazol-5(4*H*)-onato-κ^2^ *N* ^4^,*O*}nickel(II)

**DOI:** 10.1107/S1600536808009197

**Published:** 2008-04-10

**Authors:** Xin Zhang, Guo-Ying Zhang, Dan Chen, Yu-Jing Song

**Affiliations:** a College of Chemistry and Life Sciences, Tianjin Normal University, Tianjin 300074, People’s Republic of China

## Abstract

The mol­ecule of the title compound, [Ni(C_24_H_19_FN_3_O)_2_], has  twofold rotation symmetry. The Ni^II^ ion is in a square-planar coordination geometry which is distorted towards tetra­hedral and is coordinated by two N atoms of imine and two O atoms of pyrazolone from two Schiff base 4-[(*Z*)-(4-fluoro­benzyl­amino)phenyl­methyl­ene]-3-methyl-1-phenyl-1*H*-pyrazol-5(4*H*)-onate ligands.

## Related literature

For related literature, see: Sesser *et al.* (1993 ▶); Smith *et al.* (1989 ▶); Padhy *et al.* (1985 ▶); Yu *et al.* (1993 ▶); Wu *et al.* (1993 ▶); Zhao (2007 ▶); Peng *et al.* (2006 ▶); Xu *et al.* (2006 ▶); Bao *et al.* (2005 ▶); Ma *et al.* (2006 ▶); Wang (2006 ▶); Li & Wang (2007 ▶).
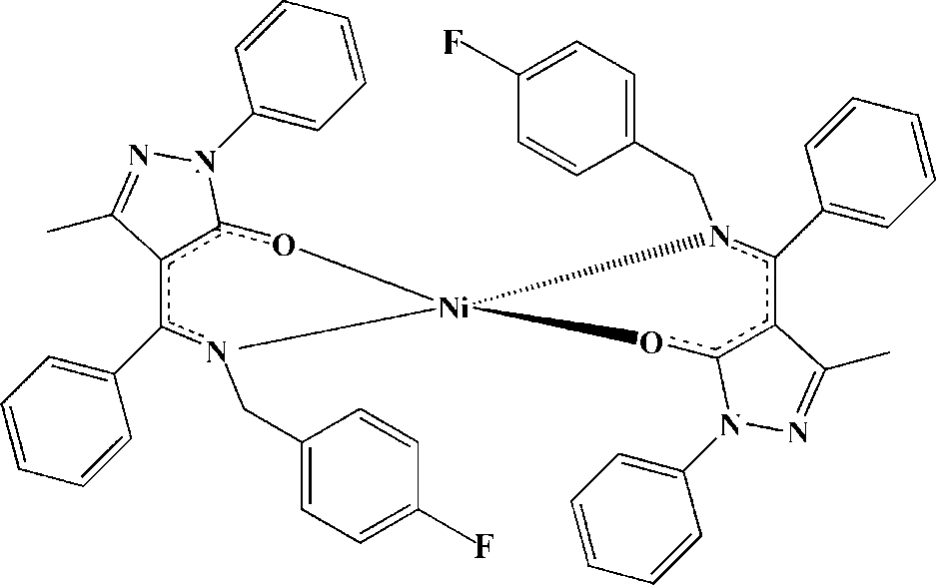


## Experimental

### 

#### Crystal data

[Ni(C_24_H_19_FN_3_O)_2_]
*M* *_r_* = 827.55
Orthorhombic, 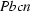

*a* = 25.475 (2) Å
*b* = 10.1620 (8) Å
*c* = 15.700 (1) Å
*V* = 4064.4 (5) Å^3^
*Z* = 4
Mo *K*α radiation
μ = 0.54 mm^−1^
*T* = 293 (2) K
0.24 × 0.22 × 0.18 mm

#### Data collection

Bruker SMART CCD diffractometer
Absorption correction: multi-scan (*SADABS*; Bruker, 1998 ▶) *T*_min_ = 0.866, *T*_max_ = 0.910
26008 measured reflections
4867 independent reflections
2916 reflections with *I* > 2σ(*I*)
*R*_int_ = 0.043

#### Refinement

*R*[*F* ^2^ > 2σ(*F* ^2^)] = 0.035
*wR*(*F* ^2^) = 0.076
*S* = 1.42
4867 reflections
268 parameters
H-atom parameters constrained
Δρ_max_ = 0.20 e Å^−3^
Δρ_min_ = −0.25 e Å^−3^

### 

Data collection: *SMART* (Bruker, 1998 ▶); cell refinement: *SAINT* (Bruker, 1998 ▶); data reduction: *SAINT*; program(s) used to solve structure: *SHELXS97* (Sheldrick, 2008 ▶); program(s) used to refine structure: *SHELXL97* (Sheldrick, 2008 ▶); molecular graphics: *SHELXTL* (Sheldrick, 2008 ▶); software used to prepare material for publication: *SHELXTL*.

## Supplementary Material

Crystal structure: contains datablocks I, global. DOI: 10.1107/S1600536808009197/lx2051sup1.cif

Structure factors: contains datablocks I. DOI: 10.1107/S1600536808009197/lx2051Isup2.hkl

Additional supplementary materials:  crystallographic information; 3D view; checkCIF report

## Figures and Tables

**Fig. 1.**
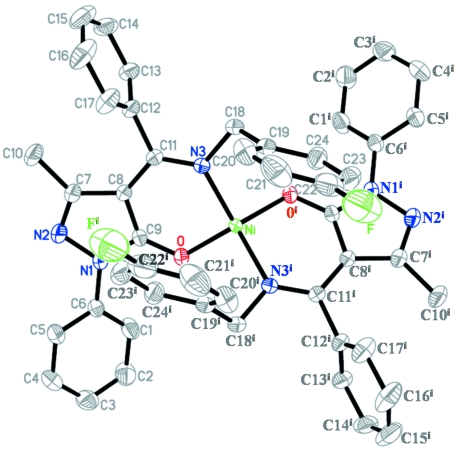
The molecular structure of (I), with anisotropic displacement ellipsoids drawn at the 30% probability level [Symmetry code: (i) -x + 1,y,-z + 1/2.]

## supplementary crystallographic information

### Comment

Complexes of Schiff bases with paramagnetic metal ions have received the much attention as a new class of potential magnetic resonance imaging (MRI) contrast agent [Sesser *et al.*, 1993; Smith *et al.*, 1989]. In addition, a great many Schiff base complexes with metals have also provoked wide interest because they possess a diverse spectrum of biological and pharmaceutical activities and catalytic properties, such as antitumor and antioxidative activities, as well as the inhibition of lipid peroxidation and so on [Padhy *et al.*, 1985; Yu *et al.*, 1993; Wu *et al.*, 1993]. In this paper, we report the synthesis and crystal structure of the title compound, (I), containing β-ketoamine ligand with organic fluorine based on pyrazolone derivatives.

The molecular structure of (I) is shown in Fig. 1. The molecule has approximate twofold rotation symmetry. The Ni^II^ ion is in a distorted square-planar coordination geometry which is different from other square-planar geometry (Wang, 2006; Li & Wang, 2007) and is coordinated by two N atoms of imine and two O atoms of pyrazoylone from two Schiff base ligands *L*. The geometry is distorted towards tetrahedral. The bond angles around Ni^II^ center range from 96.63 (5) to 146.30 (8)° and the Ni—N [1.951 (1) Å] and Ni—O [1.924 (1) Å] bond lengths in (I) are in the expected range for such complexes (Zhao, 2007; Peng *et al.*, 2006).

In the crystal structure of (I), the exocyclic C═O bond [1.284 (2) Å for C9═O1] is lengthened relative to that in the free ligand [1.252 (3) Å; Xu *et al.*, 2006], indicating the ligands in the complex have partially changed into enol form from keto form. Mean devation of 0.049 Å from the least-square plane defined by the nine constituent atoms (Ni O1 C9 N1 N2 C7 C8 C11 N3). The pyrazolone ring is nearly coplanar with the C1—C6 benzene ring and nearly perpendicular to the other two benzene rings (C12—C17 and C19–C24); the dihedral angles are 40.54 (5), 87.78 (5) and 80.99 (5)°, respectively. There are no significant intermolecular interactions in the crystal structure.

The structures of metal complexes with ligands in which the 4-fluorophenyl group of *L* is replaced by Ph in Cu(*L*^1^)_2_ (distorted square-planar coordination geometry; Bao *et al.*, 2005) and Co(*L*^2^)_2_ (distorted tetrahedral coordination geometry; Ma *et al.*, 2006) have been reported.

### Experimental

(4*Z*)-4-[(4-Fluorobenzylamino)(phenyl)-methylene]-3-methyl-1-phenyl-1*H*-pyrazol-5(4*H*)-one (1.0 mmol) and Ni(Ac)_2_ (1.0 mmol) were dissolved in MeOH (10 ml). The mixture was stirred at room temperature for about 1 h, then heated to reflux for 3 h. After allowing the solution to stand in air for 7 d, green lock-shaped crystals were formed with a yield of 40%.

### Refinement

Although all H atoms were visible in difference maps, they were placed in geometrically calculated positions, with C—H distances in the range 0.93–0.97 Å, and included in the final refinement in the riding model approximation,with *U*_iso_(H) = 1.2*U*_eq_(C) for aromatic and methylene H atoms,with *U*_iso_(H) = 1.5*U*_eq_(C) for methylic H atoms

### Crystal data

**Table:**

[Ni(C_24_H_19_FN_3_O)_2_]	*F*_000_ = 1720
*M**_r_* = 827.55	*D*_x_ = 1.352 Mg m^−^^3^
Orthorhombic, *P**b**c**n*	Mo *K*α radiation λ = 0.71073 Å
Hall symbol: -P 2n 2ab	Cell parameters from 3309 reflections
*a* = 25.475 (2) Å	θ = 2.5–22.0º
*b* = 10.1620 (8) Å	µ = 0.54 mm^−^^1^
*c* = 15.700 (1) Å	*T* = 293 (2) K
*V* = 4064.4 (5) Å^3^	BLOCK, red
*Z* = 4	0.24 × 0.22 × 0.18 mm

### Data collection

**Table:**

Bruker SMART CCD diffractometer	4867 independent reflections
Radiation source: fine-focus sealed tube	2916 reflections with *I* > 2σ(*I*)
Monochromator: graphite	*R*_int_ = 0.043
Detector resolution: 10.0 pixels mm^-1^	θ_max_ = 27.9º
*T* = 293(2) K	θ_min_ = 2.2º
φ and ω scans	*h* = −21→33
Absorption correction: multi-scan(SADABS; Bruker, 1998)	*k* = −13→12
*T*_min_ = 0.866, *T*_max_ = 0.910	*l* = −20→18
26008 measured reflections	

### Refinement

**Table:**

Refinement on *F*^2^	Secondary atom site location: difference Fourier map
Least-squares matrix: full	Hydrogen site location: inferred from neighbouring sites
*R*[*F*^2^ > 2σ(*F*^2^)] = 0.036	H-atom parameters constrained
*wR*(*F*^2^) = 0.076	*w* = 1/[σ^2^(*F*_o_^2^)] where *P* = (*F*_o_^2^ + 2*F*_c_^2^)/3
*S* = 1.42	(Δ/σ)_max_ = 0.001
4867 reflections	Δρ_max_ = 0.20 e Å^−^^3^
268 parameters	Δρ_min_ = −0.25 e Å^−^^3^
Primary atom site location: structure-invariant direct methods	Extinction correction: none

### Special details

**Table:**

Geometry. All e.s.d.'s (except the e.s.d. in the dihedral angle between two l.s. planes) are estimated using the full covariance matrix. The cell e.s.d.'s are taken into account individually in the estimation of e.s.d.'s in distances, angles and torsion angles; correlations between e.s.d.'s in cell parameters are only used when they are defined by crystal symmetry. An approximate (isotropic) treatment of cell e.s.d.'s is used for estimating e.s.d.'s involving l.s. planes.
Refinement. Refinement of *F*^2^ against ALL reflections. The weighted *R*-factor *wR* and goodness of fit *S* are based on *F*^2^, conventional *R*-factors *R* are based on *F*, with *F* set to zero for negative *F*^2^. The threshold expression of *F*^2^ > σ(*F*^2^) is used only for calculating *R*-factors(gt) *etc*. and is not relevant to the choice of reflections for refinement. *R*-factors based on *F*^2^ are statistically about twice as large as those based on *F*, and *R*- factors based on ALL data will be even larger.

### Fractional atomic coordinates and isotropic or equivalent isotropic displacement parameters (Å^2^)

**Table:**

	*x*	*y*	*z*	*U*_iso_*/*U*_eq_	
Ni	0.5000	0.74902 (3)	0.2500	0.03705 (9)	
F	0.60501 (7)	1.27896 (15)	0.15762 (10)	0.1420 (6)	
O	0.45522 (4)	0.67156 (10)	0.33499 (7)	0.0455 (3)	
N1	0.37188 (5)	0.64453 (14)	0.39135 (9)	0.0521 (4)	
N2	0.31974 (6)	0.66922 (18)	0.36965 (10)	0.0710 (5)	
N3	0.44445 (5)	0.80468 (13)	0.17239 (8)	0.0445 (3)	
C1	0.42626 (7)	0.53082 (17)	0.49505 (12)	0.0615 (5)	
H1	0.4466	0.4956	0.4514	0.074*	
C2	0.43833 (8)	0.50343 (19)	0.57895 (14)	0.0728 (6)	
H2	0.4672	0.4508	0.5915	0.087*	
C3	0.40835 (9)	0.5529 (2)	0.64395 (13)	0.0712 (6)	
H3	0.4169	0.5348	0.7003	0.085*	
C4	0.36580 (8)	0.6288 (2)	0.62491 (12)	0.0736 (6)	
H4	0.3448	0.6611	0.6686	0.088*	
C5	0.35365 (7)	0.65816 (18)	0.54168 (11)	0.0612 (5)	
H5	0.3247	0.7106	0.5296	0.073*	
C6	0.38418 (6)	0.61033 (16)	0.47621 (11)	0.0473 (4)	
C7	0.32123 (7)	0.7208 (2)	0.29336 (13)	0.0677 (6)	
C8	0.37393 (6)	0.73431 (16)	0.26211 (10)	0.0466 (4)	
C9	0.40520 (6)	0.68240 (16)	0.32787 (10)	0.0428 (4)	
C10	0.26929 (8)	0.7559 (2)	0.25261 (14)	0.1213 (12)	
H10A	0.2666	0.7134	0.1982	0.182*	
H10B	0.2672	0.8496	0.2451	0.182*	
H10C	0.2411	0.7272	0.2886	0.182*	
C11	0.39386 (6)	0.79288 (16)	0.18624 (10)	0.0446 (4)	
C12	0.35452 (6)	0.84084 (18)	0.12212 (11)	0.0498 (4)	
C13	0.33603 (7)	0.75812 (19)	0.05954 (11)	0.0587 (5)	
H13	0.3490	0.6728	0.0555	0.070*	
C14	0.29816 (7)	0.8014 (2)	0.00249 (13)	0.0735 (6)	
H14	0.2860	0.7452	−0.0399	0.088*	
C15	0.27871 (8)	0.9262 (3)	0.00842 (16)	0.0874 (8)	
H15	0.2533	0.9549	−0.0298	0.105*	
C16	0.29663 (8)	1.0086 (2)	0.07052 (18)	0.0909 (8)	
H16	0.2833	1.0936	0.0744	0.109*	
C17	0.33442 (7)	0.9671 (2)	0.12765 (14)	0.0735 (6)	
H17	0.3464	1.0240	0.1698	0.088*	
C18	0.46406 (6)	0.86510 (17)	0.09264 (10)	0.0527 (5)	
H18A	0.4346	0.8985	0.0601	0.063*	
H18B	0.4814	0.7984	0.0586	0.063*	
C19	0.50202 (7)	0.97610 (18)	0.11001 (10)	0.0507 (4)	
C20	0.48434 (9)	1.0994 (2)	0.13394 (13)	0.0776 (6)	
H20	0.4485	1.1140	0.1399	0.093*	
C21	0.51909 (13)	1.2011 (3)	0.14913 (16)	0.0981 (8)	
H21	0.5070	1.2840	0.1647	0.118*	
C22	0.57081 (12)	1.1778 (3)	0.14100 (14)	0.0883 (8)	
C23	0.59040 (9)	1.0606 (3)	0.11648 (14)	0.0788 (7)	
H23	0.6264	1.0477	0.1113	0.095*	
C24	0.55516 (7)	0.95964 (19)	0.09921 (12)	0.0612 (5)	
H24	0.5678	0.8791	0.0799	0.073*	

### Atomic displacement parameters (Å^2^)

**Table:**

	*U*^11^	*U*^22^	*U*^33^	*U*^12^	*U*^13^	*U*^23^
Ni	0.02468 (14)	0.06300 (18)	0.02347 (13)	0.000	0.00016 (12)	0.000
F	0.1785 (15)	0.1505 (13)	0.0970 (12)	−0.1026 (12)	−0.0011 (10)	−0.0095 (10)
O	0.0322 (6)	0.0664 (7)	0.0379 (7)	0.0036 (6)	0.0040 (5)	0.0071 (6)
N1	0.0325 (8)	0.0818 (10)	0.0421 (9)	−0.0017 (7)	0.0053 (6)	0.0073 (8)
N2	0.0316 (9)	0.1299 (15)	0.0514 (11)	−0.0065 (9)	0.0032 (7)	0.0108 (10)
N3	0.0349 (8)	0.0695 (9)	0.0291 (7)	−0.0051 (7)	−0.0005 (6)	0.0011 (7)
C1	0.0614 (13)	0.0680 (13)	0.0551 (13)	0.0088 (10)	0.0174 (9)	0.0123 (11)
C2	0.0678 (14)	0.0820 (15)	0.0686 (15)	0.0128 (11)	0.0094 (12)	0.0304 (12)
C3	0.0771 (16)	0.0885 (15)	0.0480 (13)	−0.0108 (12)	0.0019 (11)	0.0194 (11)
C4	0.0771 (16)	0.0996 (16)	0.0442 (13)	0.0030 (13)	0.0142 (10)	−0.0034 (12)
C5	0.0516 (12)	0.0838 (14)	0.0482 (12)	0.0107 (10)	0.0091 (9)	0.0030 (10)
C6	0.0410 (10)	0.0609 (11)	0.0400 (11)	−0.0061 (9)	0.0066 (8)	0.0055 (8)
C7	0.0327 (10)	0.1215 (18)	0.0488 (12)	−0.0031 (10)	−0.0004 (9)	0.0073 (12)
C8	0.0312 (8)	0.0730 (12)	0.0357 (11)	−0.0037 (8)	−0.0012 (7)	0.0015 (9)
C9	0.0341 (9)	0.0583 (10)	0.0359 (10)	−0.0038 (8)	0.0052 (8)	−0.0020 (8)
C10	0.0306 (11)	0.260 (4)	0.0731 (16)	−0.0052 (15)	−0.0036 (10)	0.0410 (19)
C11	0.0332 (9)	0.0644 (11)	0.0360 (10)	0.0005 (8)	−0.0043 (7)	−0.0042 (8)
C12	0.0321 (10)	0.0721 (13)	0.0452 (11)	−0.0058 (9)	−0.0051 (8)	0.0063 (10)
C13	0.0461 (11)	0.0822 (13)	0.0479 (11)	−0.0086 (10)	−0.0115 (8)	0.0055 (11)
C14	0.0585 (14)	0.1094 (17)	0.0527 (13)	−0.0275 (12)	−0.0213 (10)	0.0174 (12)
C15	0.0522 (14)	0.113 (2)	0.097 (2)	−0.0190 (14)	−0.0315 (12)	0.0466 (16)
C16	0.0561 (14)	0.0820 (16)	0.134 (2)	0.0067 (11)	−0.0280 (14)	0.0224 (16)
C17	0.0502 (13)	0.0788 (14)	0.0915 (17)	0.0026 (11)	−0.0198 (11)	−0.0045 (12)
C18	0.0408 (10)	0.0854 (13)	0.0319 (10)	−0.0046 (9)	−0.0045 (8)	0.0085 (9)
C19	0.0462 (11)	0.0744 (12)	0.0316 (9)	−0.0046 (10)	−0.0041 (8)	0.0125 (8)
C20	0.0710 (15)	0.0945 (17)	0.0675 (16)	−0.0001 (13)	0.0161 (11)	−0.0024 (13)
C21	0.130 (2)	0.0836 (16)	0.0808 (19)	−0.0205 (17)	0.0294 (17)	−0.0168 (14)
C22	0.108 (2)	0.104 (2)	0.0527 (14)	−0.0481 (19)	−0.0053 (14)	0.0039 (14)
C23	0.0555 (14)	0.1039 (18)	0.0770 (17)	−0.0236 (13)	−0.0132 (11)	0.0407 (15)
C24	0.0466 (12)	0.0723 (13)	0.0646 (13)	−0.0031 (10)	−0.0003 (9)	0.0268 (11)

### Geometric parameters (Å, °)

**Table:**

Ni—O^i^	1.924 (1)	C10—H10B	0.9600
Ni—O	1.924 (1)	C10—H10C	0.9600
Ni—N3^i^	1.951 (1)	C11—C12	1.502 (2)
Ni—N3	1.951 (1)	C12—C13	1.376 (2)
F—C22	1.373 (2)	C12—C17	1.384 (2)
O—C9	1.284 (2)	C13—C14	1.388 (2)
N1—C9	1.365 (2)	C13—H13	0.9300
N1—N2	1.394 (2)	C14—C15	1.365 (3)
N1—C6	1.412 (2)	C14—H14	0.9300
N2—C7	1.308 (2)	C15—C16	1.364 (3)
N3—C11	1.313 (2)	C15—H15	0.9300
N3—C18	1.481 (2)	C16—C17	1.382 (3)
C1—C6	1.375 (2)	C16—H16	0.9300
C1—C2	1.381 (2)	C17—H17	0.9300
C1—H1	0.9300	C18—C19	1.511 (2)
C2—C3	1.370 (3)	C18—H18A	0.9700
C2—H2	0.9300	C18—H18B	0.9700
C3—C4	1.364 (3)	C19—C24	1.374 (2)
C3—H3	0.9300	C19—C20	1.383 (3)
C4—C5	1.376 (2)	C20—C21	1.381 (3)
C4—H4	0.9300	C20—H20	0.9300
C5—C6	1.378 (2)	C21—C22	1.345 (3)
C5—H5	0.9300	C21—H21	0.9300
C7—C8	1.436 (2)	C22—C23	1.347 (3)
C7—C10	1.513 (3)	C23—C24	1.390 (3)
C8—C9	1.407 (2)	C23—H23	0.9300
C8—C11	1.425 (2)	C24—H24	0.9300
C10—H10A	0.9600		
			
O^i^—Ni—O	131.70 (6)	H10B—C10—H10C	109.5
O^i^—Ni—N3^i^	96.99 (5)	N3—C11—C8	121.8 (1)
O—Ni—N3^i^	96.63 (5)	N3—C11—C12	121.0 (1)
O^i^—Ni—N3	96.63 (5)	C8—C11—C12	117.27 (14)
O—Ni—N3	96.99 (5)	C13—C12—C17	119.0 (2)
N3^i^—Ni—N3	146.30 (8)	C13—C12—C11	120.6 (2)
C9—O—Ni	119.5 (1)	C17—C12—C11	120.4 (2)
C9—N1—N2	111.3 (1)	C12—C13—C14	120.4 (2)
C9—N1—C6	128.4 (1)	C12—C13—H13	119.8
N2—N1—C6	119.1 (1)	C14—C13—H13	119.8
C7—N2—N1	105.6 (1)	C15—C14—C13	120.2 (2)
C11—N3—C18	120.6 (1)	C15—C14—H14	119.9
C11—N3—Ni	125.6 (1)	C13—C14—H14	119.9
C18—N3—Ni	113.79 (9)	C14—C15—C16	119.9 (2)
C6—C1—C2	119.8 (2)	C14—C15—H15	120.1
C6—C1—H1	120.1	C16—C15—H15	120.1
C2—C1—H1	120.1	C15—C16—C17	120.6 (2)
C3—C2—C1	120.8 (2)	C15—C16—H16	119.7
C3—C2—H2	119.6	C17—C16—H16	119.7
C1—C2—H2	119.6	C12—C17—C16	120.0 (2)
C4—C3—C2	119.2 (2)	C12—C17—H17	120.0
C4—C3—H3	120.4	C16—C17—H17	120.0
C2—C3—H3	120.4	N3—C18—C19	111.9 (1)
C3—C4—C5	120.6 (2)	N3—C18—H18A	109.2
C3—C4—H4	119.7	C19—C18—H18A	109.2
C5—C4—H4	119.7	N3—C18—H18B	109.2
C6—C5—C4	120.3 (2)	C19—C18—H18B	109.2
C6—C5—H5	119.8	H18A—C18—H18B	107.9
C4—C5—H5	119.8	C24—C19—C20	117.7 (2)
C1—C6—C5	119.2 (2)	C24—C19—C18	121.2 (2)
C1—C6—N1	121.4 (2)	C20—C19—C18	121.1 (2)
C5—C6—N1	119.5 (2)	C21—C20—C19	121.0 (2)
N2—C7—C8	112.2 (2)	C21—C20—H20	119.5
N2—C7—C10	117.2 (2)	C19—C20—H20	119.5
C8—C7—C10	130.6 (2)	C22—C21—C20	118.7 (2)
C9—C8—C11	124.6 (2)	C22—C21—H21	120.7
C9—C8—C7	104.1 (2)	C20—C21—H21	120.7
C11—C8—C7	131.2 (2)	C21—C22—C23	123.1 (2)
O—C9—N1	122.0 (2)	C21—C22—F	118.2 (3)
O—C9—C8	131.2 (2)	C23—C22—F	118.8 (3)
N1—C9—C8	106.8 (1)	C22—C23—C24	118.0 (2)
C7—C10—H10A	109.5	C22—C23—H23	121.0
C7—C10—H10B	109.5	C24—C23—H23	121.0
H10A—C10—H10B	109.5	C19—C24—C23	121.5 (2)
C7—C10—H10C	109.5	C19—C24—H24	119.3
H10A—C10—H10C	109.5	C23—C24—H24	119.3
			
O^i^—Ni—O—C9	−111.23 (12)	C11—C8—C9—N1	175.72 (15)
N3^i^—Ni—O—C9	143.24 (12)	C7—C8—C9—N1	−0.71 (18)
N3—Ni—O—C9	−5.86 (12)	C18—N3—C11—C8	179.54 (14)
C9—N1—N2—C7	0.5 (2)	Ni—N3—C11—C8	−0.8 (2)
C6—N1—N2—C7	168.80 (16)	C18—N3—C11—C12	−0.4 (2)
O^i^—Ni—N3—C11	137.19 (14)	Ni—N3—C11—C12	179.28 (12)
O—Ni—N3—C11	3.64 (14)	C9—C8—C11—N3	−1.0 (3)
N3^i^—Ni—N3—C11	−109.53 (14)	C7—C8—C11—N3	174.36 (17)
O^i^—Ni—N3—C18	−43.14 (11)	C9—C8—C11—C12	178.89 (16)
O—Ni—N3—C18	−176.69 (11)	C7—C8—C11—C12	−5.7 (3)
N3^i^—Ni—N3—C18	70.14 (10)	N3—C11—C12—C13	90.9 (2)
C6—C1—C2—C3	1.0 (3)	C8—C11—C12—C13	−89.0 (2)
C1—C2—C3—C4	0.7 (3)	N3—C11—C12—C17	−92.2 (2)
C2—C3—C4—C5	−1.4 (3)	C8—C11—C12—C17	87.9 (2)
C3—C4—C5—C6	0.4 (3)	C17—C12—C13—C14	0.6 (3)
C2—C1—C6—C5	−2.0 (3)	C11—C12—C13—C14	177.48 (16)
C2—C1—C6—N1	177.55 (16)	C12—C13—C14—C15	−0.4 (3)
C4—C5—C6—C1	1.3 (3)	C13—C14—C15—C16	0.1 (3)
C4—C5—C6—N1	−178.28 (17)	C14—C15—C16—C17	0.1 (4)
C9—N1—C6—C1	−43.5 (3)	C13—C12—C17—C16	−0.4 (3)
N2—N1—C6—C1	150.35 (16)	C11—C12—C17—C16	−177.32 (19)
C9—N1—C6—C5	136.03 (18)	C15—C16—C17—C12	0.1 (4)
N2—N1—C6—C5	−30.1 (2)	C11—N3—C18—C19	126.69 (16)
N1—N2—C7—C8	−0.9 (2)	Ni—N3—C18—C19	−53.00 (16)
N1—N2—C7—C10	179.03 (17)	N3—C18—C19—C24	104.07 (18)
N2—C7—C8—C9	1.1 (2)	N3—C18—C19—C20	−78.6 (2)
C10—C7—C8—C9	−178.9 (2)	C24—C19—C20—C21	−2.1 (3)
N2—C7—C8—C11	−175.04 (18)	C18—C19—C20—C21	−179.47 (19)
C10—C7—C8—C11	5.0 (4)	C19—C20—C21—C22	−0.6 (4)
Ni—O—C9—N1	−171.42 (12)	C20—C21—C22—C23	1.8 (4)
Ni—O—C9—C8	6.3 (2)	C20—C21—C22—F	−178.94 (19)
N2—N1—C9—O	178.37 (15)	C21—C22—C23—C24	−0.2 (4)
C6—N1—C9—O	11.4 (3)	F—C22—C23—C24	−179.45 (17)
N2—N1—C9—C8	0.20 (19)	C20—C19—C24—C23	3.7 (3)
C6—N1—C9—C8	−166.79 (16)	C18—C19—C24—C23	−178.87 (16)
C11—C8—C9—O	−2.2 (3)	C22—C23—C24—C19	−2.7 (3)
C7—C8—C9—O	−178.65 (18)		

Symmetry codes: (i) −*x*+1, *y*, −*z*+1/2.
